# The use of upadacitinib for refractory pityriasis rubra pilaris: A case report

**DOI:** 10.1016/j.jdcr.2025.09.031

**Published:** 2025-10-10

**Authors:** Tajauna Batchelor, Clayton D. Conner

**Affiliations:** a University of Pikeville – Kentucky College of Osteopathic Medicine, Pikeville, Kentucky; b Dermatology Associates of Northern Kentucky, Florence, Kentucky

**Keywords:** biologics, immune dermatology, inflammatory dermatosis, JAK inhibitors, pityriasis rubra pilaris, upadacitinib

## Introduction

Pityriasis rubra pilaris (PRP) is a rare inflammatory skin disorder characterized by scaly erythematous papules and plaques with well-demarcated “islands of sparing”.^1^ Of its 6 subtypes, Type I (classic adult-onset) is the most common and can resemble psoriasis or atopic dermatitis clinically. Follicular hyperkeratotic papules and orange, waxy palmoplantar keratoderma help differentiate PRP from other papulosquamous conditions, but its underlying causes remain poorly understood, which makes selecting safe and effective treatments challenging.^1^

Treatment options include emollients, topical corticosteroids, systemic retinoids, broad-spectrum immunosuppressants, and biologics targeting tumor necrosis factor-alpha, interleukin (IL)-12/23, or IL-17.^2^ However, many patients experience only partial or temporary relief with current treatment options.^2^^,^^3^ Thus, the lack of standardized guidelines underscores the need for alternative therapies in recalcitrant cases.

This report presents a case of treatment-resistant PRP that achieved rapid and sustained remission with upadacitinib, a selective Janus kinase (JAK) 1 inhibitor. This case adds to the growing evidence supporting the use of JAK inhibitors in PRP.

## Case presentation

A 57-year-old man presented with a 27-month history of a progressively worsening pruritic eruption, initially limited to his palms before rapidly spreading to his trunk and extremities over several weeks. His medical history included well-controlled hypertension, originally treated with lisinopril, which was later switched to losartan to exclude drug-related causes. He denied any personal or family history of psoriasis, atopic dermatitis, or other inflammatory dermatoses.

**Fig 1:**
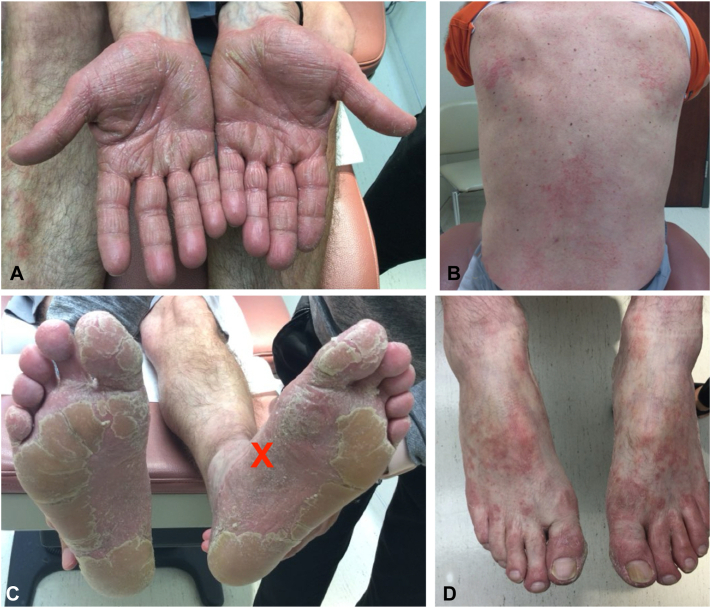
**A-D,** Erythroderma with prominent scaling on the patient’s palms and soles at initial presentation, with “islands of sparing” evident on the back and feet. A red “X” marks the biopsy site on the left instep. (Photos taken in September 2022).

**Fig 2:**
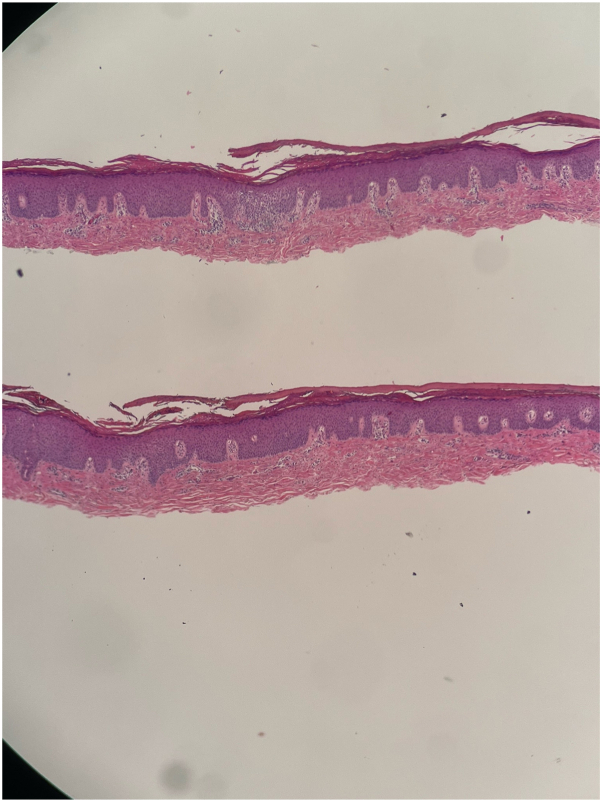
Left instep shave biopsy (September 21, 2022) consistent with pityriasis rubra pilaris. The section shows hyperkeratosis with alternating orthokeratosis and parakeratosis, thickened rete ridges and suprapapillary plates. This is also focal spongiosis and a superficial perivascular lymphocytic infiltrate. (H&E, original magnification ×10).

On examination, there were well-demarcated orange, waxy keratoderma on the palms and soles. Large red-orange hyperkeratotic plaques with scattered areas of unaffected skin (“islands of sparing”) were observed on the back and feet (Fig 1). A shave biopsy from the left instep (marked by the red X) showed alternating orthokeratosis and parakeratosis, thickened rete ridges and suprapapillary plates consistent with PRP (Fig 2). The patient was screened with standard laboratory testing in anticipation of needing systemic immunosuppressants, systemic retinoids, and biologics, which showed no contraindications to therapies.

Initial treatment included topical clobetasol ointment and over-the-counter emollients, which provided limited relief. The rash and pruritus cleared with a 3-week prednisone taper starting at 60 mg daily and a 6-week course of cyclosporine 200 mg daily, but flared 2 weeks after discontinuation.

He was concurrently treated with infliximab 5 mg/kg every 8 weeks, dapsone 50 mg daily, and methotrexate increased to 25 mg weekly. These therapies nearly cleared the disease, with mild remaining involvement on the palms. Unfortunately, this effect diminished after about 3 months. Biologic therapy was initiated with ixekizumab 80 mg monthly following a psoriasis loading dose schedule but was discontinued after 3 months due to no clinical response.

**Fig 3:**
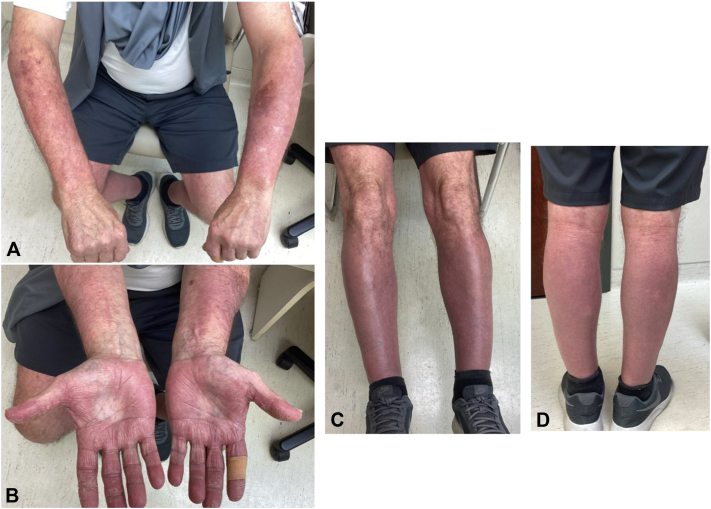
**A-D,** Recurrence of PRP 11.5 months after initiating ustekinumab therapy. Diffuse erythematous plaques with scale have returned on the hands, feet, and distal extremities.

Next, ustekinumab 90 mg was administered every 12 weeks, following a psoriasis loading dose schedule, which resulted in nearly complete clearance that lasted 11.5 months. However, the patient developed rapidly progressing lesions on the hands, feet, and distal extremities (Fig 3) with no obvious trigger or prodrome, which led him to schedule an urgent follow-up appointment.

**Fig 4:**
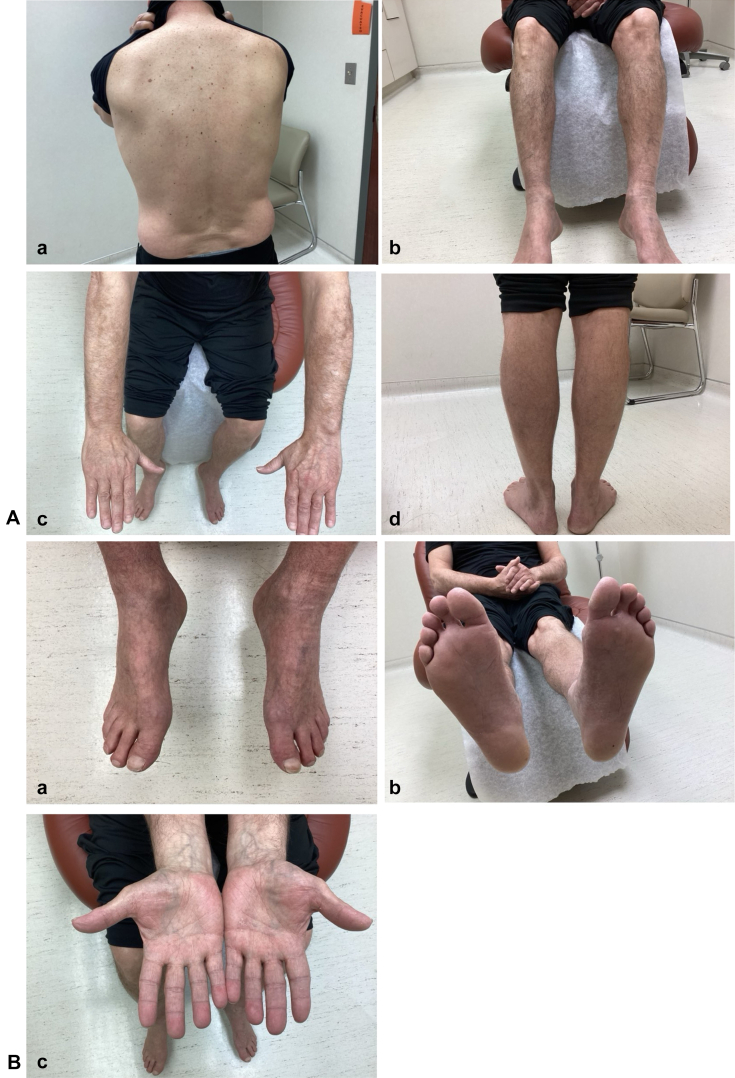
**A,** panels **a-d,** and **B,** panels **a-c,** Near-complete clearance of the skin 2 weeks after initiating upadacitinib 30 mg daily. The patient’s previously affected areas show resolved erythema and scaling with postinflammatory hyperpigmentation.

At the follow-up on 10/9/2024, upadacitinib 30 mg daily was initiated in place of another prednisone taper. After 2 weeks, he achieved nearly complete clearance (Fig 4). When titrating the dose to 15 mg daily, symptoms and rash returned within days. Resuming upadacitinib at 30 mg daily led to almost immediate resolution of lesions and symptoms. Ten months later, he remains clear without side effects and plans to continue his current treatment.

## Discussion

Due to its heterogeneous presentation and poorly understood pathophysiology, PRP is diagnostically and therapeutically challenging. Recent studies propose overlapping immunologic mechanisms with psoriasis and Familial PRP (Type V), particularly involving the IL-17 and IL-23 signaling pathways.^4^^,^^5^ These cytokines are regulated through the JAK signal transducer and activator of transcription signaling pathway,^6^ offering a potential therapeutic target in refractory cases.

The JAK/signal transducer and activator of transcription pathway stands out for its broad immunomodulatory effects and selectivity for specific enzymes within the JAK family. Upadacitinib, a selective JAK1 inhibitor, has demonstrated efficacy in various immune-mediated diseases, including atopic dermatitis and psoriatic arthritis.^6^^,^^7^ In this case, upadacitinib induced rapid clearance with sustained remission of symptoms at 30 mg daily after failing multiple systemic and biologic therapies. The return of symptoms when attempting to reduce dosage to 15 mg and subsequent resolution after returning to 30 mg daily further underscores its pivotal role in disease control.

Low prevalence and diverse clinical presentations of PRP pose significant challenges to early recognition and standardization of care, especially in patients with skin of color, where diagnostic delays may contribute to prolonged morbidity.^8^ Given the evolving understanding of PRP and its shared immunologic profile with other inflammatory conditions, further studies are needed to evaluate long-term safety, efficacy, and optimal dosing strategies for JAK inhibitors in this patient population.

Reports on the use of JAK inhibitors in PRP remain limited. This case highlights key clinical features of PRP, supports consideration of JAK inhibitors in treatment-resistant cases, and contributes to the expanding literature on the management of recalcitrant disease.

## Conclusion

Upadacitinib may be a safe and effective option for PRP that is refractory to conventional and biologic therapies.

### Declaration of generative AI and AI-assisted technologies in the writing process

During the preparation of this work, the author used AI to improve the readability and structure of the paper. After using this tool/service, the author reviewed and edited the content as needed and takes full responsibility for the content of the publication.

## Conflicts of interest

None disclosed.
